# Complicated Sigmoid Diverticulitis Mimicking Pelvic Pain in a Patient With Giant Uterine Fibroids: A Case Report

**DOI:** 10.7759/cureus.77979

**Published:** 2025-01-25

**Authors:** Rosa Miranda Thais, Mina Sarofim, Andrew Gilmore

**Affiliations:** 1 Department of Colorectal Surgery, Liverpool Hospital, Liverpool, AUS; 2 School of Medicine, University of New South Wales, Sydney, Kensington, AUS

**Keywords:** colonic perforation, complicated diverticulitis, diverticular disease of the colon, diverticulosis, herniation, intestinal microbiota, large fibroids, surgical management, uterine fibroid

## Abstract

This report describes the case of a woman in her late forties with a history of large uterine fibroids who presented to the Emergency Department with colicky suprapubic pain and urinary symptoms. Initial computed tomography (CT) showed uncomplicated acute diverticulitis but despite antibiotic treatment, her condition worsened, and a follow-up CT revealed sigmoid perforation, requiring high anterior resection and subtotal hysterectomy. The case highlights how large fibroids exert external pressure on the colon, impairing motility and increasing the risk of diverticula formation. Alterations in the gut microbiome may contribute to colonic mucosal inflammation, commonly seen in diverticular disease. Patients with uterine fibroids have altered microbiome composition, which could further increase the risk of diverticular disease. These findings provide a pathway for future research into the influence of uterine fibroids on the pathophysiology of diverticular disease.

## Introduction

Diverticular disease is a common gastrointestinal condition, typically affecting the sigmoid colon. The herniation of the colonic mucosa and submucosa through the muscle layer causes the formation of diverticula, a condition that primarily affects older individuals in developed countries [1]. Diverticular disease can present as diverticulosis or progress to acute diverticulitis, which requires medical management. In some cases, diverticulitis leads to more severe complications, such as perforation, abscess, or fistula formation, which necessitate surgical intervention [2].

Uterine fibroids are benign neoplasms of the uterus that commonly affect women of reproductive age. Large fibroids, particularly those located posteriorly, can exert pressure on the colon and rectum, potentially affecting bowel motility and increasing colonic pressure, which may contribute to the formation of diverticula. Due to their bulk, giant fibroids often cause symptoms of bowel and bladder dysfunction [3,4]. The relationship between uterine fibroids and gastrointestinal disorders, particularly diverticular disease, remains poorly defined in the literature.

This report presents a rare instance of a patient with significantly large uterine fibroids who presented with vague pelvic pain and developed complicated diverticulitis, requiring emergency surgery. This case highlights the potential impact of fibroid size and location on gastrointestinal function and contributes to the limited understanding of the interactions between gynaecological and gastrointestinal conditions. It underscores the importance of considering coexisting conditions in diagnosing patients with overlapping symptoms.

## Case presentation

A woman in her 40s with a history of multiple large uterine fibroids was referred to a tertiary colorectal surgery unit with colicky suprapubic pain for the past five days, associated with dysuria and urinary frequency radiating across the lower abdomen. On examination, she was febrile with a distended abdomen and a large tender palpable mass up to the mid epigastrium. Laboratory parameters showed mild leucocytosis (11.1 x10^9^/L) and C-reactive protein (CRP) of 105 mg/L.

**Figure 1:**
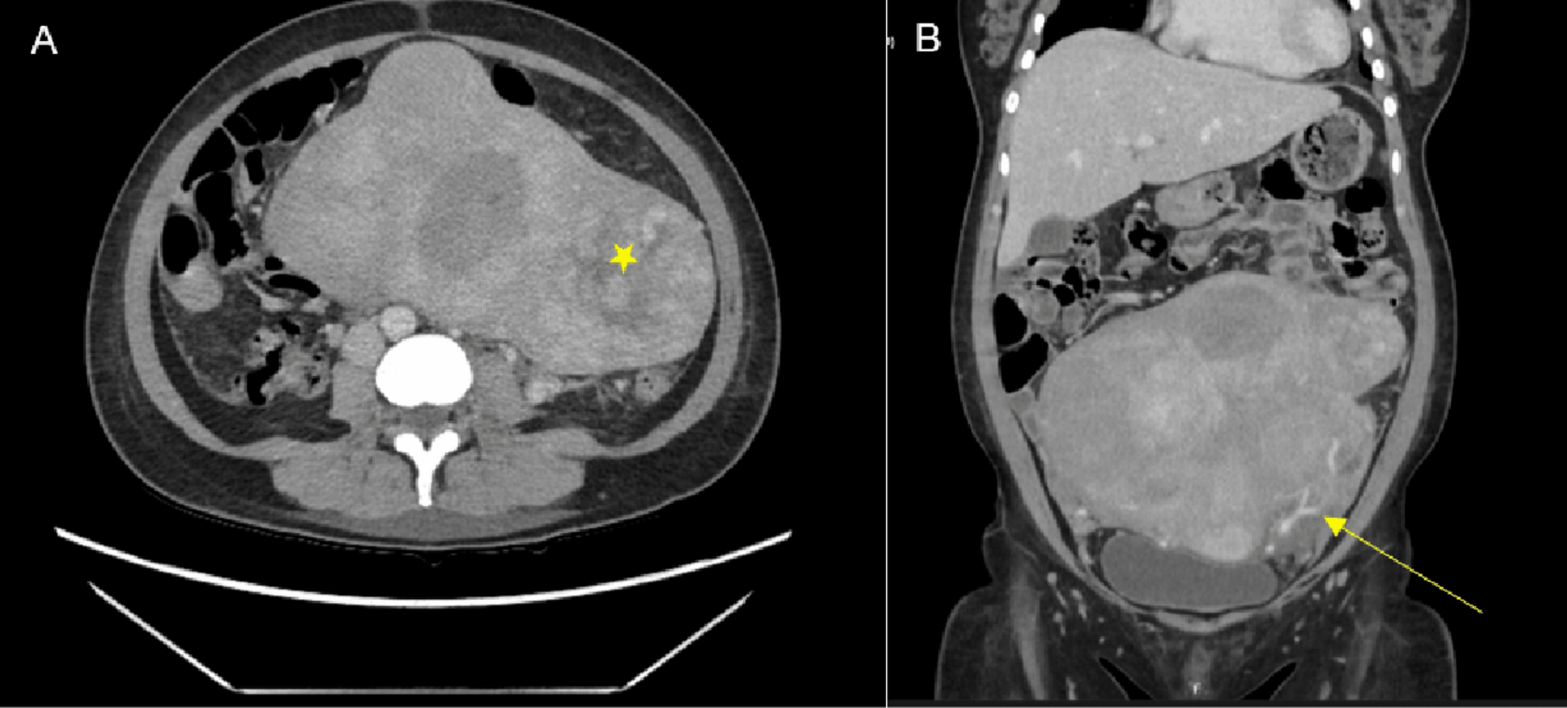
Computed tomography of the abdomen (A axial, B coronal) demonstrating giant fibroids (*) and colonic stranding (arrow) of diverticulitis.

Computer tomography (CT) of the abdomen and pelvis showed the presence of a multi-fibroid uterus with the largest transaxial dimension measuring 20x12 cm, in addition to mural thickening of the proximal and mid-sigmoid colon, pericolic fat stranding consistent with uncomplicated acute diverticulitis (Figure 1). The patient was treated with intravenous antibiotics following local guidelines (ampicillin, gentamicin, and metronidazole). She remained febrile despite five days of treatment and an antibiotic upgrade (piperacillin-tazobactam). Inflammatory markers showed persistent leucocytosis 13 x10^9^/L and a CRP of 95 mg/L. Progress CT showed progression to sigmoid perforation with localized free gas.

The patient was operatively managed with a subtotal hysterectomy, on-table colonic lavage, and high restorative anterior resection. She made an uneventful recovery and was discharged on postoperative day 8. Histopathology confirmed perforated diverticulitis with abscess formation. The uterus showed proliferative endometrium and benign leiomyomata.

## Discussion

Complicated diverticulitis in patients with large fibromyomas is rarely reported in the literature. Impaired gut motility secondary to extrinsic colonic pressure and gut dysbiosis are essential but underrecognized risk factors for developing diverticular disease in these patients. Steward et al. reported that patients with large fibroids experience bladder and bowel dysfunction as well as abdominal protrusion and pelvic pain [4]. According to Violi et al., bowel motility is associated with neural degeneration, producing uncoordinated contractions and high-pressure-producing diverticulosis [5]. In our patient, the extremely large multi-fibroid uterus is likely to have caused extrinsic compression, which impaired colonic motility.

There is growing research focusing on the role of gut microbiota in the pathogenesis of multiple gastrointestinal diseases, including diverticular disease [6,7]. Gut dysbiosis is considered a decrease in microbial diversity and an increase in species that promote multiple inflammatory pathways, compromising the mucosal barrier and the local immune function [8].

Recent studies have shown that patients with uterine fibroids have altered gut microbiome composition, ecological network, and functionality compared to healthy individuals [9]. According to Mao et al. [10] and Piccioni et al. [11], uterine fibroids are associated with the diminished prevalence of Lactobacilli and other 'good bacteria,' essential in preventing inflammatory mediators' release. Additionally, there is an increased presence of pathogenic 'harmful bacteria' such as *Enterobacteriaceae*, *Streptococcus*, and *Bacteroides*, which may explain why those with fibroids have an increased incidence of developing acute diverticulitis compared to healthy individuals.

However, the role of the microbiome in the development of acute diverticulitis needs to be better understood, partly due to the heterogeneity of studies and small sample sizes. Reitano et al., in their systematic review of eight studies, found that most were case-control or cohort studies, with only one study including patients with acute diverticulitis [12]. Similarly, Cameron et al.'s systematic review, which included 12 studies, found no significant difference in microbiota diversity between control samples in nine studies [8].

This case emphasizes the importance of considering coexisting conditions such as uterine fibroids in patients with diverticular disease. It also highlights the need for thorough diagnostic evaluation and closer monitoring of the condition in cases where the clinical presentation is atypical or when multiple pathologies are suspected.

There are limitations to this report. Its single-case design makes it difficult to draw broad conclusions. There is no robust literature on the role of the microbiome in acute diverticulitis and the optimal management of complicated diverticulitis in patients with extremely large fibroids. As demonstrated in our patient, a period of non-operative management was trialled, but after no improvement, surgical resection and primary anastomosis were performed with good postoperative recovery.

Future research should explore the role of uterine fibroids in the pathophysiology of diverticular disease. More extensive cohort studies and prospective trials are needed to determine the prevalence of diverticular disease in patients with large uterine fibroids and whether the location and size could increase the risk of complications. This could aid in ensuring appropriately timed operative management.

## Conclusions

Pelvic pain in patients with large uterine fibroids could obscure the presence of underlying gastrointestinal conditions, such as acute diverticulitis, making diagnosis more challenging. This case highlights a rare but essential connection between large uterine fibroids and complicated diverticulitis. It reminds clinicians to consider both pathologies when evaluating patients with overlapping symptoms. A level of suspicion should be maintained in patients with fibroid-associated pelvic pain as surgical management may be required on an individualised basis.
